# Multispectral snapshot imaging of skin microcirculatory hemoglobin oxygen saturation using artificial neural networks trained on *in vivo* data

**DOI:** 10.1117/1.JBO.27.3.036004

**Published:** 2022-03-26

**Authors:** Maria Ewerlöf, Tomas Strömberg, Marcus Larsson, E. Göran Salerud

**Affiliations:** Linköping University, Department of Biomedical Engineering, Linköping, Sweden

**Keywords:** multispectral imaging, artificial neural networks, hemoglobin oxygen saturation, skin microcirculation, diffuse reflectance spectroscopy

## Abstract

**Significance:**

Developing algorithms for estimating blood oxygenation from snapshot multispectral imaging (MSI) data is challenging due to the complexity of sensor characteristics and photon transport modeling in tissue. We circumvent this using a method where artificial neural networks (ANNs) are trained on *in vivo* MSI data with target values from a point-measuring reference method.

**Aim:**

To develop and evaluate a methodology where a snapshot filter mosaic camera is utilized for imaging skin hemoglobin oxygen saturation ($S_{O_{2}}$), using ANNs.

**Approach:**

MSI data were acquired during occlusion provocations. ANNs were trained to estimate $S_{O_{2}}$ with MSI data as input, targeting data from a validated probe-based reference system. Performance of ANNs with different properties and training data sets was compared.

**Results:**

The method enables spatially resolved estimation of skin tissue $S_{O_{2}}$. Results are comparable to those acquired using a Monte-Carlo-based approach when relevant training data are used.

**Conclusions:**

Training an ANN on *in vivo* MSI data covering a wide range of target values acquired during an occlusion protocol enable real-time estimation of $S_{O_{2}}$ maps. Data from the probe-based reference system can be used as target despite differences in sampling depth and measurement position.

## 1 Introduction

Skin microcirculation is noninvasively accessible using optical instrumentation, and its physiologically related parameters, such as hemoglobin oxygen saturation ($S_{O_{2}}$), red blood cell (RBC) tissue fraction, and melanin content can be monitored using diffuse reflectance spectroscopy (DRS).^1^ Most DRS systems consist of a broadband light source and a spectrally resolving detector, although spectral resolution can also be achieved by sweeping or tuning the light source emission spectra. For fiber-based systems, the source–detector distance is small, typically in the mm range, causing detected photons to traverse sub-mm to single mm in tissue depth depending on the wavelength range. For imaging systems, a slightly larger sampling depth can be assumed in the visible wavelength range.^2^ Regardless of modality, the detected backscattered light intensity is affected by tissue scattering and absorption optical properties. This effect is wavelength dependent and, hence, DRS contains information about concentrations of biological chromophores^3^^,^^4^ dominated by oxy- and deoxyhemoglobin, melanin, and bilirubin. Analyzing the backscattered diffuse spectra, DRS can estimate microcirculatory physiological parameters such as $S_{O_{2}}$ without having to assess intensity variations associated with arterial blood pulsations, as is done in pulse oximetry.^1^

Until recently, many DRS systems were fiber-based and limited to pointwise measurements. Recent advancements in multispectral imaging (MSI) technology have created possibilities to register spatially resolved images, where each pixel is spectrally resolved, a so-called data cube. This can be acquired either by sweeping the light source and/or detector over an imaged area, collecting one row of spectral data at a time, or by sweeping through the spectral dimension using optical filters.^5^ These methods are limited by sweep time when it comes to studying rapidly changing events. There are snapshot systems capable of instantaneous capture of a whole spectral data cube using on-chip optical filters placed in a repeating mosaic pattern. These systems overcome the temporal limitation but introduce new challenges due to a limited spectral resolution with complex filter characteristics.^6^

Measured DRS spectra reflect changes in both absorption and scattering and are often analyzed by fitting to an analytical or numerical model of skin diffuse reflectance to assess the microcirculatory status of skin tissue.^7^ Solving the inverse problem involves iterative updates of model parameters until measured and modeled spectra agree. Diffusion theory (DT) is an approximation valid for simplified geometries and some other assumptions. This theory allows for analytical calculation of DRS spectra given the optical and geometrical properties of the measurement set-up.^8^^–^^12^ A more generally applicable model that is considered the golden standard is Monte Carlo (MC) simulations of light transport.^13^^–^^18^ With MC, statistical sampling of millions or billions of photon trajectories is performed until a desired accuracy is obtained. Different methods have been proposed to accelerate MC simulations,^19^ some employing graphics processing units (GPUs). Recently, a real-time analysis using inverse MC for pointwise measurements has been achieved, calculating 2 to 10 sets of microcirculation parameters per second on a lap-top with a built-in GPU.^20^ For imaging applications, where the amount of data vastly surpasses that of a pointwise technique, processing is much more demanding.^2^

To avoid computationally demanding search algorithms, some research groups have applied machine learning approaches to directly extract tissue properties.^14^^,^^21^^,^^22^ The use of an artificial neural network (ANN) for determining optical properties was first reported by Farrell et al., where the algorithm was trained using DT.^21^ Later Farrell et al. evaluated ANNs on phantom and *in vivo* data.^23^ Since then, ANNs have demonstrated in several publications to accurately estimate the optical properties^21^^,^^24^^–^^26^ and $S_{O_{2}}$.^20^^,^^27^ The computational efficiency of machine learning algorithms makes them well suited for real-time imaging applications, where the calculation load can be exactly determined by the number of inputs and number of nodes in the ANN.

Relevant and representative training data is of outmost importance when using ANNs. It must cover the full range of optical properties, types of tissues, measurement sites, etc., that may be targeted by the application. Training data may be generated using forward MC simulations when determining optical properties from reflectance measurements^25^^,^^28^^,^^29^ or chromophore concentrations directly.^20^^,^^27^ This approach requires an accurate model of the system properties such as spectral sensitivity, noise, and possible drift as well as a model of light propagation in tissue, and the measurement setup (skin type, location, etc.). Training data for snapshot imaging sensors must take the complex characteristics of the filter mosaic into account. However, we know from experience that it is difficult to determine the filter characteristics with such accuracy that reliable training data can be produced from MC simulations.^2^ Another approach, which to our knowledge has not previously been applied, is to train the algorithm using *in vivo* data that was coregistered with a validated reference system. This eliminates the need for a calibration procedure to handle the hardware characteristics and extensive light transport theory modeling.

The aim of this study was to develop and evaluate a methodology using a fast ANN method trained on *in vivo* data to estimate skin $S_{O_{2}}$ images from data captured by a snapshot MSI camera. The target data were concurrently measured by a validated point-measuring optical spectroscopy system. Measurements were continuously performed on healthy subjects during arterial and venous occlusion and release provocations, to capture $S_{O_{2}}$ values covering almost the complete 0% to 100% range. As a first proof-of-principle, this study is conducted in a population with a limited range of skin tones, focusing on demonstrating how variations in forearm skin $S_{O_{2}}$ can be imaged.

## 2 Materials and Methods

The experimental setup and data acquisition were previously described in Ewerlöf et al. 2017^30^ and 2021^2^ and are summarized in the following sections. In this paper, the model used for analysis of MSI data is changed from inverse Monte Carlo to ANNs.

### 2.1 Equipment

Diffuse reflectance data were acquired by a snapshot MSI camera (xiSpec MQ0022HG-IM-SM4X4-VIS, XIMEA^®^, Germany) containing a Fabry–Perot interference filter array (4×4  pixels) arranged in a recurring mosaic pattern. The 16 different filters have complex spectral sensitivities in the wavelength range 450 to 650 nm and have substantial spectral overlap. The mosaic filter array was positioned on top of the CMOS sensor (2048×1088  pixels), optically aligned to the CMOS sensor pixels. The camera was equipped with a 16-mm lens (C Series VIS-NIR Lens, Edmund Optics Inc., Barrington) and mounted side-by-side to an 8 LED ring light (R130, Smart Vision Lights) emitting light in the range 390 to 765 nm. A crossed polarizer/analyzer pair was placed in front of the light source (polarizer) and the detecting MSI camera (analyzer) to avoid specular reflections. Data were collected by the MSI system using MATLAB (MATLAB version 8.2.0, 2013b, computer software, The MathWorks Inc., Natick, Massachusetts). Sixteen consecutive snapshots, each with exposure time 20 ms, were acquired from the MSI system and summed to reduce noise and amount of data, resulting in an effective framerate of 1.4 frames per second. A PF-6000 EPOS instrument (Perimed AB, Järfälla, Sweden) was used as reference system.^1^ The probe-based method measures diffuse reflectance spectra at two source–detector fiber separations (0.4- and 1.2-mm center–center separation) in the wavelength interval 450 to 850 nm with a framerate of 1 Hz. Spectra were analyzed using an inverse Monte Carlo algorithm based on a three-layered tissue model that has been shown to assess absolute values of $S_{O_{2}}$ (%) to within 5% root-mean-square (RMS) deviation.^31^ The light source of the EPOS system was an AvaLight-HAL-S (Avantes, Apeldorn, The Netherlands) for the first 11 subjects and was changed to an AvaLight-HAL-S-Mini for the remaining 13 subjects. The arm cuff used for occlusion was controlled by the EPOS system.

### 2.2 Measurement Protocols

Two separate occlusions (arterial and venous occlusion) were performed on 24 healthy subjects with a previously described protocol.^2^ Measurements were performed in seated position with one arm resting on a vacuum pillow (Germa Protec, AB Germa, Kristianstad, Sweden) for support and fixation. Left or right arm was chosen randomly and both protocols were performed on the same arm, starting with arterial occlusion. An arm cuff was placed around the upper arm and the subject was acclimatized in the room (24°C to 26°C room temperature) for 15 min before measurements started. For spatial reference in the region imaged by the MSI camera 3×3 ink dots were painted on the skin to mark, the corners of a quadrant of four 2×2  cm regions of interest (ROIs). The MSI camera was positioned 30 cm above the surface of the arm. The EPOS probe was applied to the skin using double-adhesive tape avoiding visible vessels, positioned distally and close to the area marked by the ink dots. The probe was repositioned between provocations.

For the arterial occlusion protocol, the subject’s forearm was completely occluded (arterial occlusion) by inflating the arm cuff to 250 mmHg for 5 min followed by a rapid release of pressure, causing reactive hyperemia reperfusion of the lower arm tissue. After a resting period of at least 45 min, the arm cuff was inflated once more, this time to 45 mmHg, causing a 5-min venous occlusion of the same arm. The venous occlusion was followed by a rapid release of pressure, again causing reperfusion of the lower arm tissue. Data were acquired for 5 min before occlusion (baseline), 5 min during occlusion, and for 10 min after release (reperfusion) for both protocols.

### 2.3 Study Subjects

Measurements were performed on 24 healthy subjects and data from 20 of those were included in this study (10 women and 10 men, aged 21 to 39 years). They all had skin type I to III, self-reported using Fitzpatrick skin typing test.^32^ Exclusion criteria were known skin conditions or circulatory diseases, use of medication affecting the circulatory system and smoking. Extensive physical activity was not allowed for 24 h and coffee for 4 h prior to measurements. Written informed consent was obtained from all participants. The study was approved by the Regional Ethical Review Board in Linköping (D. No. 2015/392-31).

### 2.4 Data Exclusion

Data from four subjects were excluded due to exclusion criteria (one subject had coffee prior to the measurements and one had a skin condition), nonadherence to the study protocol (one subject) and poor EPOS signal (one person). For the remaining subjects, data collected when the light source was turned off during dark measurements were excluded, as well as data from a few erroneous EPOS registrations.

### 2.5 Data Preprocessing

Each 2048×1088-pixel image from the MSI camera contains intensity data shaped by the 16 unique bandpass filters overlaid on the sensor. Before further analysis, these raw images were dark-corrected by subtracting a dark raw image acquired interleaved during each measurement series. A multispectral data cube was then reconstructed using a weighted bilinear interpolation demosaicing algorithm^33^^,^^34^ followed by spatial binning of 4×4  pixels for each wavelength band. This resulted in a multispectral data cube with an effective resolution of 512×272  pixels for each of the 16 wavelength bands. Finally, the data cubes where white normalized using a multispectral data cube collected from a white reference Spectralon^®^ tile (Labsphere, Inc., North Sutton, New Hampshire). The acquisition of dark raw images and white calibration raw images is further described in Ref. 2.

### 2.6 Selection of Data

A 9-min time interval (from 1 min before occlusion until 3 min after release) covering the full dynamic range of $S_{O_{2}}$ values was selected for all subjects, involving about 760 acquired MSI data cubes each for arterial and venous occlusion. The location of the four ROIs was continuously tracked for spatial reference during each measurement, as described in Sec. 2.5 in Ref. 2. Each pixel of the acquired data cube provided MSI intensities from 16 channels forming the spectrum used as training data. Each spectrum was normalized to its mean intensity. To increase the amount of training data and to minimize spatial dependencies, spectral data from 10 pixels in each data cube were randomly chosen from one ROI near the EPOS probe. This resulted in about 7600×2=15,200 spectra acquired from the two occlusions for each subject. When comparing results to the previously reported inverse Monte Carlo algorithm,^2^ the mean spectrum over the 2×2  cm ROI was used. For imaging purposes, the whole area of interest (the lower arm) was masked out from the background and each pixel analyzed separately.

**Fig. 1:**
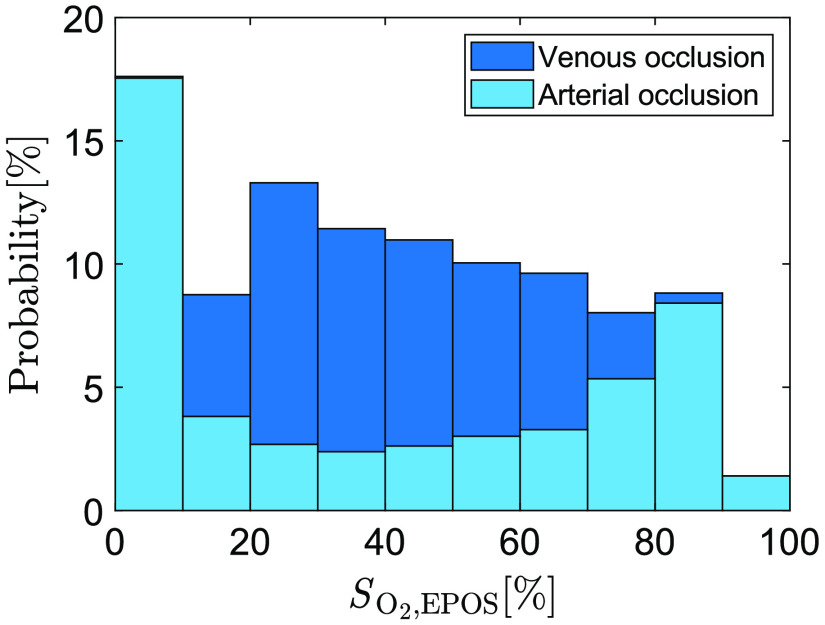
The combined distribution of $S_{O_{2},\mathrm{EPOS}}$ values used as targets for training ANNs (all 20 subjects). Light blue bars show values acquired during arterial occlusion and dark blue represent values acquired during venous occlusion.

The target $S_{O_{2}}$ values measured by the EPOS reference system ($S_{O_{2},\mathrm{EPOS}}$) during arterial and venous occlusion were unevenly distributed, as shown in Fig. 1. By including both provocations, a more even distribution between 0% and 100% was achieved.

### 2.7 Training of ANNs

Training of shallow ANNs (one hidden layer) was performed using the deep learning toolbox in MATLAB (MATLAB version 9.10.0, R2021a). ANNs were trained with MSI data (intensities from 16 wavelength bands) as input, using $S_{O_{2},\mathrm{EPOS}}$ as target. Each ANN was initialized using the function *fitnet* where the number of hidden layers was stated, and the network training function set to Levenberg–Marquardt backpropagation using mean-square-error loss function. The hyperbolic tangent function was used as activation function of the nodes in the hidden layer. Using default settings, the input data for training were divided into training data (70%), validation data (15%), and test data (15%). The ANNs were trained with the MATLAB function *train* using parallel computing. Training parameters were set to default values. Before repeating the training, the ANN was re-initialized with new weight and bias values to avoid similar starting set-ups.

**Fig. 2:**
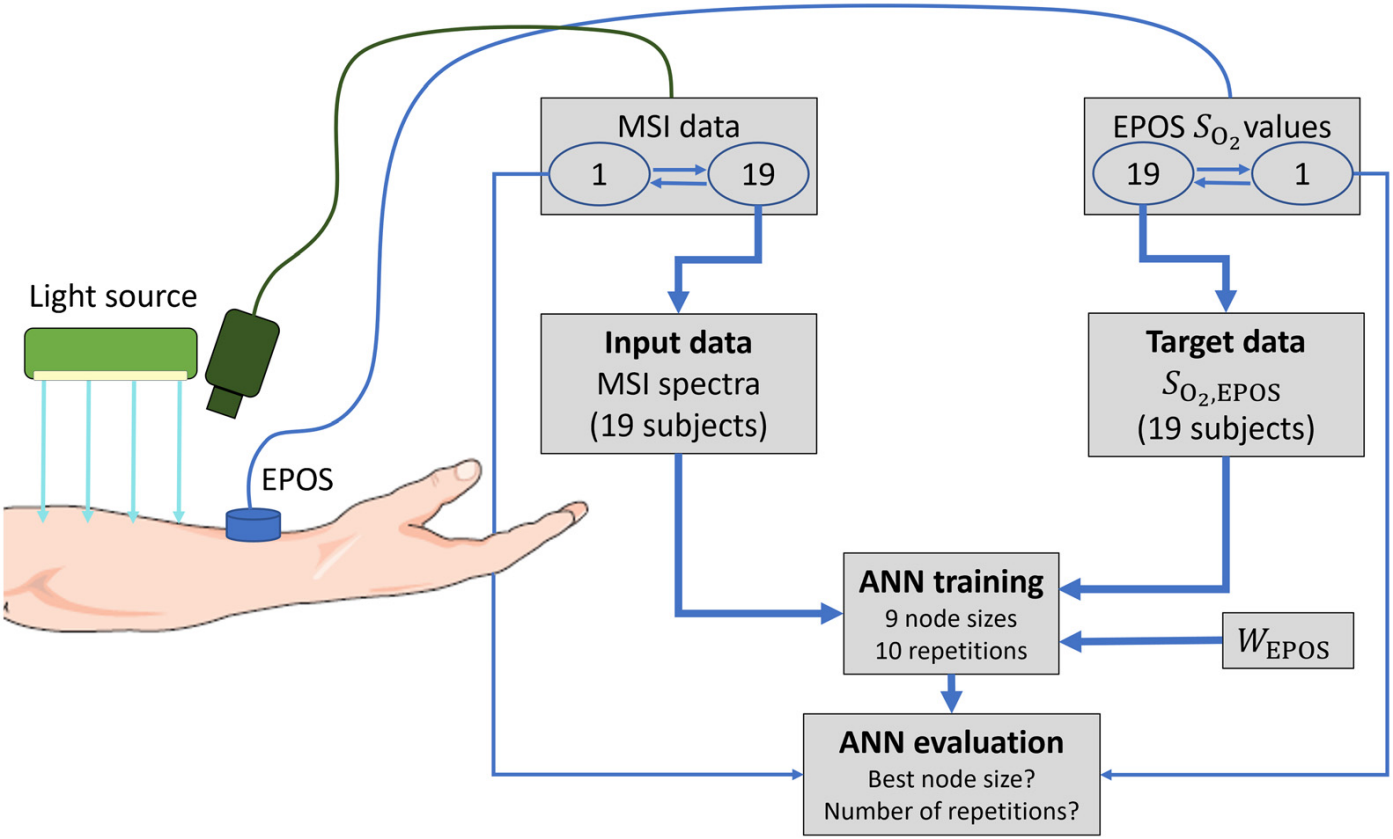
An overview of the experiment set-up and main steps of training and evaluation procedures. Each training data set consisted of data from 19 out of 20 subjects. Data from the left-out subject, iteratively chosen, were used for evaluation.

For the ANN training and evaluation, the principle of leave-one-subject-out cross-validation^35^ was applied, i.e., data from all subjects except one were used for training, validation, and testing. Untouched and independent data from the left-out subject were then used for the final evaluation of the ANN. A set contained about 19×15,200=288,800 spectra used for training, validation, and testing, while 15,200 unused spectra were used for the final evaluation. This procedure was repeated 20 times until all subjects had been used in the final evaluation. For each left-out subject, training was repeated 10 times with different starting parameters for each set of training data and for nine different sizes of the hidden layer (1, 2, 3, 4, 5, 7, 10, 15, and 20 nodes) resulting in 90 trained ANNs. The concurrently measured $S_{O_{2},\mathrm{EPOS}}$ was used as target for each spectrum. A summary of the training procedure is shown in Fig. 2.

The target $S_{O_{2},\mathrm{EPOS}}$ values of the training data were unevenly distributed in the interval 0% to 100%, as shown in Fig. 1. To account for this, error weights were calculated and used for training and evaluation. For the entire data set (data from all 20 subjects), the number of targets were binned in their respective 10% interval. Error weights were calculated as 1/number of targets in each bin and normalized to a number between 0 and 1. The resulting array, W, containing a weight for each of the 10% bins, was applied to the data sets to level out the impact of data from the respective bin.

To investigate the importance of including data with a full span of $S_{O_{2}}$ target values, training was performed without using data from arterial occlusion. This training was performed using the same W as described above. The trained ANNs were evaluated for data from arterial occlusion protocols including both lower and higher $S_{O_{2}}$ values than those included in the training data set.

### 2.8 Evaluation of ANN Results

The performance of a trained ANN was represented by a weighted RMS error, $E_{\mathrm{rms},W}$, between target values from the EPOS system, $S_{O_{2},\mathrm{EPOS}}$, and values estimated by ANN, $S_{O_{2},\mathrm{ANN}}$, calculated according to Eq. (1). $W_{\mathrm{EPOS}}$ was a vector containing the corresponding weight from W for each target value:

$$E_{\mathrm{rms},W}=\sqrt{\frac{\sum W_{\mathrm{EPOS}}\cdot {\left(S_{O_{2},\mathrm{EPOS}}-S_{O_{2},\mathrm{ANN}}\right)}^{2}}{\sum W_{\mathrm{EPOS}}}}.$$ (1)

ANNs chosen for each set of training data were applied to the 20 evaluation data sets containing data from arterial occlusion and to averaged data over a 2×2  cm ROI analyzed by the inverse MC algorithm presented in Ref. 2. To illustrate application of the ANN method for imaging, spatially resolved $S_{O_{2}}$ images, where a trained ANN was applied to spectral data from individual pixels, were also calculated for three points of time (during baseline, at end of occlusion, and during reperfusion).

### 2.9 Statistics

Bland–Altman analysis^36^ was performed on the results to compare ANN results to target EPOS values. The analysis was performed for three time intervals: baseline (from 60 s before until 5 s before occlusion starts), end of occlusion (from 60 s before until 10 s before occlusion ends), and early after release (from 5 s before until 55 s after release). For the first two intervals, mean values were calculated for ANN and EPOS methods, respectively. During release the increase in $S_{O_{2}}$ is distinct. Since we wanted to capture the peak during release, we chose to use the median of nine samples around the peak EPOS value in the chosen interval. Mean bias was calculated, and upper and lower limits set to mean ±1.96 standard deviations (SD).

## 3 Results

**Fig. 3:**
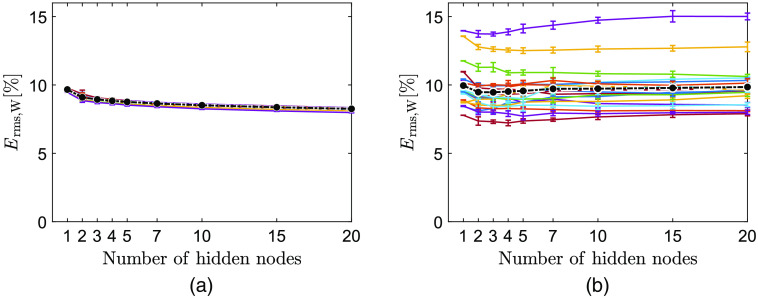
Calculated $E_{\mathrm{rms},W}$ for ANNs trained with varying number of hidden nodes and applied to (a) the test data and (b) the leave-one-subject-out evaluation data, respectively. Each line represents the average ANN performance for 10 repetitions of training. Error bars show SD. Black dash-dotted line indicates the mean $E_{\mathrm{rms},W}$ over all data sets for each node size, respectively.

The performance of the trained ANNs during both training (a) and evaluation (b) for the 20 different data sets is shown in Fig. 3. For training, all data sets show a conformance in $E_{\mathrm{rms},W}$, while it varies more when the trained ANNs are applied to unknown evaluation data. The number of nodes only had a minor influence on $E_{\mathrm{rms},W}$. Mean $E_{\mathrm{rms},W}$ over all 20 evaluation data sets showed a lowest value for three nodes (9.5%). The variation in performance between 10 repeated trainings (shown as error bars) lies within 0.1% when calculated for data used during training and 0.7% for evaluation data. Therefore, the following results use only the first repetition of ANNs trained using three nodes in the hidden layer.

**Fig. 4:**
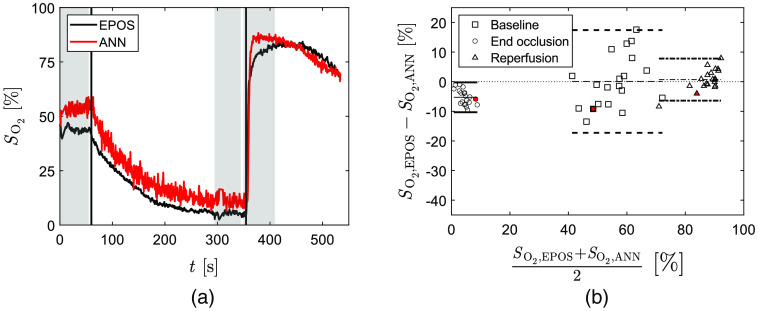
(a) Typical individual $S_{O_{2},\mathrm{ANN}}$ (red) and corresponding $S_{O_{2},\mathrm{EPOS}}$ (black) during arterial occlusion. Vertical lines indicate start and end off occlusion. Time intervals used for Bland–Altman analysis are marked in gray for baseline (0 to 55 s), end of occlusion (300 to 350 s), and reperfusion (355 to 410 s). (b) Agreement between $S_{O_{2},\mathrm{ANN}}$ and $S_{O_{2},\mathrm{EPOS}}$ as a Bland–Altman analysis of 20 subjects at baseline (squares/dashed lines), end of occlusion (circles/solid lines), and reperfusion (triangles/dash-dotted lines). The mean difference for each phase is represented by thin lines and the difference ±1.96*SD (95% prediction interval) by bold lines. Symbols filled with red represent data from the dataset in (a).

The time-resolved $S_{O_{2},\mathrm{ANN}}$ for the evaluation data set with median $E_{\mathrm{rms},W}$ (9.1%) is shown in Fig. 4(a) together with corresponding $S_{O_{2},\mathrm{EPOS}}$. Bland–Altman analysis was performed for the 20 evaluation data sets analyzed with their respective trained ANN at three time intervals indicated by gray in Fig. 4(a). The result is shown in Fig. 4(b). ANNs give results similar to EPOS values in baseline with mean bias 0.08% with upper and lower limits of 17.4% and −17.3% deviation and at reperfusion with mean bias 0.7% with upper and lower limits of −7.8% and −6.4% deviation. Results at end of occlusion show a larger difference with mean bias −5.3% with upper and lower limits of −0.2% and −10.3% deviation, respectively.

**Fig. 5:**
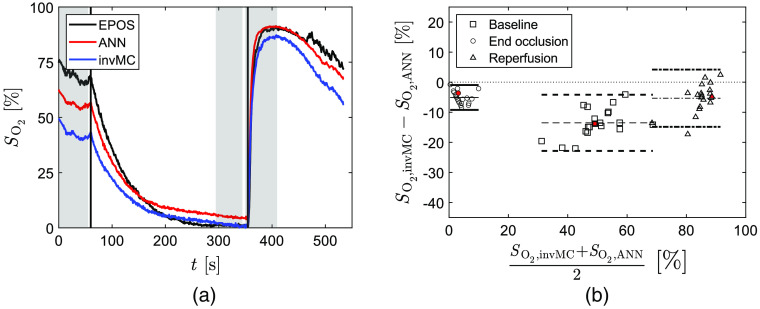
(a) The $S_{O_{2}}$ during arterial occlusion estimated by the inverse MC algorithm in Ref. 2 ($S_{O_{2},\mathrm{invMC}}$, blue), by ANN ($S_{O_{2},\mathrm{ANN}}$, red) as well as corresponding reference value ($S_{O_{2},\mathrm{EPOS}}$, black). Vertical lines indicate start and end off occlusion. Time intervals used for Bland–Altman analysis are marked in gray for baseline (0 to 55 s), end of occlusion (300 to 350 s), and reperfusion (355 to 410 s). (b) Agreement between $S_{O_{2},\mathrm{ANN}}$ and $S_{O_{2},\mathrm{invMC}}$ for the 20 subjects at baseline (squares/dashed lines), end of occlusion (circles/solid lines), and reperfusion (triangles/dash-dotted lines). The mean difference for each phase is represented by thin lines and the difference ±1.96×SD (95% prediction interval) by bold lines. Symbols filled with red represent data from the data set in (a).

The ANNs were applied to the same MSI data analyzed by inverse MC in Ref. 2. The time-resolved result for a representative data set is shown in Fig. 5(a) and compared to the inverse MC result, $S_{O_{2},\mathrm{invMC}}$, and $S_{O_{2},\mathrm{EPOS}}$ target data. In this case, the ANN method, compared to the inverse MC algorithm, gives a result closer to EPOS reference values in baseline and an even better match at reperfusion. At end of occlusion, the inverse MC algorithm is closer to EPOS. The Bland–Altman analysis comparing the inverse MC algorithm to ANN results for the 20 sets of ROI data in Fig. 5(b) shows higher values for the latter in all time intervals on average. The mean bias is −13.5% with upper and lower limits of −4.2% and −23.0% deviation in baseline, −5.1% with upper and lower limits of −1.0% and −9.2% deviation at end of occlusion, and −5.3% with upper and lower limits of 4.2% and −14.9% deviation for reperfusion.

**Fig. 6:**
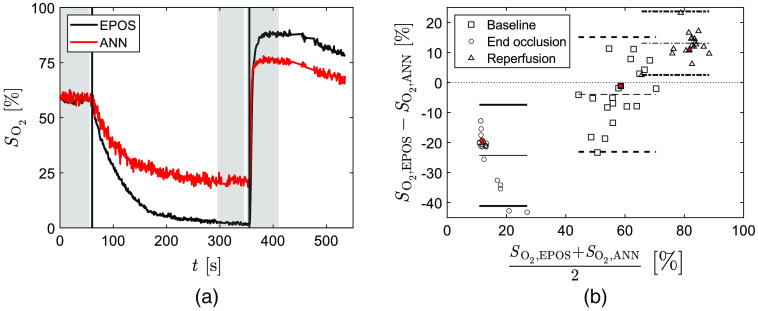
(a) Typical individual $S_{O_{2},\mathrm{ANN}}$ (red), when ANNs were trained using only venous occlusion data, and corresponding $S_{O_{2},\mathrm{EPOS}}$ (black). Vertical lines indicate start and end off occlusion. Time intervals used for Bland–Altman analysis are marked in gray for baseline (0 to 55 s), end of occlusion (300 to 350 s), and reperfusion (355 to 410 s). (b) Agreement between $S_{O_{2},\mathrm{ANN}}$ and $S_{O_{2},\mathrm{EPOS}}$ for the 20 subjects at baseline (squares/dashed lines), end of occlusion (circles/solid lines), and reperfusion (triangles/dash-dotted lines). The mean difference for each phase is represented by thin lines and the difference ±1.96×SD (95% prediction interval) by bold lines. Symbols filled with red represent data from the data set in (a).

Results from training including only data from venous occlusion, $S_{O_{2},\mathrm{ANN},\mathrm{venOccl}}$, is shown in Fig. 6(a) for a representative evaluation data set and clearly show the restricted ANN’s difficulties to estimate both low and high target $S_{O_{2},\mathrm{EPOS}}$ values. Bland–Altman analysis in Fig. 6(b) confirms the observation. The mean bias in baseline is −4.0% with upper and lower limits of 15.1% and −23.1% deviation. At end of occlusion, the mean bias is −24.3% with upper and lower limits of −7.4% and −41.1% deviation and results at reperfusion show a mean bias of 13.1% with upper and lower limits of 23.7% and 2.5% deviation, respectively.

Table 1 presents the average $S_{O_{2}}$ for the three time intervals indicated by gray in Fig. 4(a) seen over all 20 evaluation data sets when estimated by EPOS, ANNs applied on evaluation data, ANNs and inverse MC algorithm applied on ROI data, and the ANNs trained on venous occlusion data applied on data from arterial occlusion.

**Table 1:** $S_{O_{2}}$ estimated by ANN and EPOS for arterial occlusion. Mean (SD) and [max min] are given at baseline, end of occlusion, and start of reperfusion. The $S_{O_{2},\mathrm{ANN}}$ is calculated for evaluation MSI data and for the mean over ROI-data. The result for the case where ANNs were trained only on data from venous occlusion is presented in the last row.

	Baseline	End of occlusion	Reperfusion
$S_{O_{2},\mathrm{ANN}}$ (%)^a^	55.8 (7.1)	7.1 (2.9)	87.3 (3.6)
	[40.3 74.9]	[1.9 12.7]	[75.2 91.0]
$S_{O_{2},\mathrm{ANN}}$ (%)^b^	56.6 (7.1)	6.5 (2.8)	87.6 (3.7)
	[41.0 75.8]	[0.8 11.0]	[75.2 91.5]
$S_{O_{2},\mathrm{ANN},\mathrm{venOccl}}$ (%)^a^	59.9 (6.0)	26.1 (8.3)	74.9 (4.6)
	[46.3 71.5]	[17.7 48.6]	[64.3 83.6]
$S_{O_{2},\mathrm{EPOS}}$ (%)	55.9 (10.5)	1.8 (1.6)	88.0 (6.2)
	[39.0 72.0]	[-0.5 5.3]	[66.8 96.2]
$S_{O_{2},\mathrm{invMC}}$ (%)^b^	43.1 (9.5)	1.4 (2.3)	82.2 (6.6)
	[21.4 61.6]	[0.0 8.9]	[61.6 92.7]

a Mean value over 10 pixels calculated from MSI data.

b Calculated from MSI data (mean intensity over ROI).

**Fig. 7:**
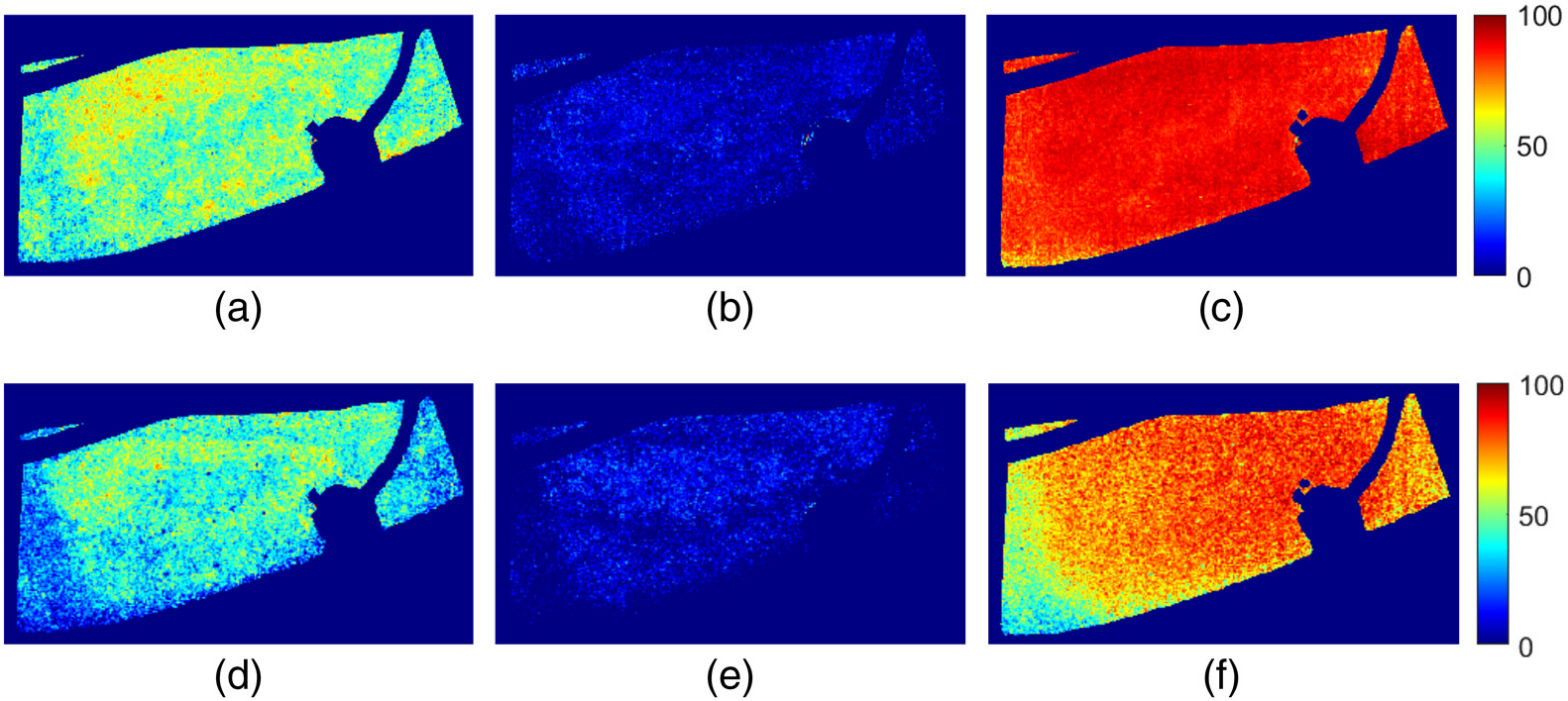
Spatially resolved $S_{O_{2}}$ estimated from data analyzed by (a)–(c) a trained ANN and (d)–(f) by the inverse MC algorithm described in Ref. 2. Data for three timepoints during arterial occlusion are presented: (a), (d) during baseline; (b), (e) end of occlusion; and (c), (f) reperfusion.

Our proposed and trained ANN was applied pixelwise to three intensity data cubes, resulting in $S_{O_{2},\mathrm{ANN}}$ maps from three temporal points (during baseline, end of occlusion, and reperfusion) for arterial occlusion. The results are shown in Figs. 7(a)–7(c) together with the corresponding $S_{O_{2},\mathrm{invMC}}$ maps (d)–(f). Both algorithms show distinct differences for the three time points with $S_{O_{2}}$ values around 50% during baseline, close to 0% at end of occlusion and values between 90% and 100% for reperfusion. The computational time for an image (512×270  pixels) using a three node ANN was 0.056 s. For comparison, a 10 node ANN spent 0.066 s and an ANN with 20 nodes 0.082 s for the same image. The inverse MC algorithm took on average 0.051 s per pixel which adds up to 1 h 58 min for a 512×270-pixel image. Computations for both methods were performed by a stationary computer with an Intel^®^ Xeon^®^ CPU E5-1620 processor and 16 GB RAM.

## 4 Discussion

In this study, we investigated if data acquired *in vivo* from healthy subjects can be used for training ANNs that reliably estimate $S_{O_{2}}$ images from MSI data. Target data were $S_{O_{2}}$ values from a validated point measuring EPOS system. Evaluation with independent MSI data not included in the training data set, i.e., data from the left-out subject, showed excellent conformance between ANN estimates and EPOS values. Data used for training were measured by the same system and under similar conditions as the evaluation data, which eliminates the need to account for the system’s specific characteristics such as impinging light source spectrum, sensor filters, and polarization. The utilized xiSpec MSI camera has a sensor with complex transmission characteristics for each of the 16 different bandpass filters. Every xiSpec sensor is unique in terms of sensitivity to different wavelengths and training is consequently camera specific. Therefore, our presented method can be applied to other types of MSI systems if training data are acquired by the same system used for measurements.

Other groups have used training data generated with MC simulations of tissue models.^7^^,^^27^ This approach does not need a series of *in vivo* measurements, but instead needs modeling of both measurement system and light transport in the tissue under study. The noise induced during measurements by camera, light source and/or aspects of tissue and light transport model affects measured spectra and must be considered and accurately modelled.^20^ In our case noise is inherent from the *in vivo* measurements. The randomly chosen pixels in every measurement handle possible differences over the sensor area. Zherebtsov et al. use spectra simulated from a three-layered tissue model as training data with oxygen saturation ranging between 30% and 100%.^7^ Their ANN was then applied on spectra collected from the palm side of the hand during a 3-min finger occlusion protocol and a spectral range 675 to 825 nm was used for oxygen saturation estimations. Reported estimated $S_{O_{2}}$ values were 83% and 62% for baseline and at end of occlusion, respectively. Our corresponding values are 56% and 7% from ANN estimates and 56% and 2% measured with EPOS on the volar forearm. Differences might partly be explained by a difference in vessel beds with higher oxygenation in finger tissues. We investigated this further in a pilot experiment with a 5-min arterial finger occlusion using EPOS on the palm side of a finger. The results for $S_{O_{2}}$ ranged from 75% in baseline to ∼0% at end of occlusion. The longer wavelengths used by Zherebtsov et al. may give a larger penetration depth, but they report a value of ∼2  mm in tissue simulating phantoms. Light transport simulations using Monte Carlo technique show a sampling depth of 1.7 mm at 630 nm in skin tissue for our MSI system.^2^ Therefore, the differences probably origin from the lack of training data for low $S_{O_{2}}$ values, and small distinct spectral variations with oxygen saturation in their chosen wavelength range. From presented best-fit spectra their overall goodness-of-fit appear great, but finer distinct spectral variations (e.g., the 540 to 580 nm range and the 758 nm absorption peak) that are uniquely related to the oxygen saturation level is insufficiently accounted for in their inverse modeling. The high $S_{O_{2}}$ at end occlusion is hence likely an effect of an inverse solution that focuses more on the overall shape rather than the finer details. This demonstrates the importance of having accurate models that fully capture what is being measured by hyperspectral imaging or MSI when performing simulations.

Regardless of how training data are acquired, it must be relevant for the anticipated application and cover the full dynamics of possible outcomes. The arterial occlusion provocation provides a wide range of $S_{O_{2}}$ values from 0% at end of occlusion to values close to 100% during the reperfusion phase. Venous occlusion affects the $S_{O_{2}}$, but not to the same extent and mainly provide data in the range 10% to 70%. Applying ANNs trained on data from only venous occlusion on evaluation data from arterial occlusion show difficulties for ANNs to find accurate $S_{O_{2}}$ values for data with low and high target values, since those were not included in the training data set. Therefore, it is strongly recommended to include data from both arterial and venous occlusion in the training data and limit the impact of the still slightly uneven distribution of $S_{O_{2}}$ values by adding the error weight vector, W. One additional reason for including results from venous occlusion provocations in the training data set was to add data collected with varying levels of RBC tissue fraction.

Data originating from the same subject will not be completely independent as the optical properties (e.g., scattering and absorption) within one subject only vary to some extent. Similarly, samples from one subject, taken at the same time point, but at different spatial locations, will not be completely independent as the local microcirculatory blood volume and blood oxygenation will only vary slightly, governed by the overall tissue status. We try to overcome this partly by randomly sampling different spatial points over time and partly using two different provocations where blood amount and oxygenation will vary over time. Still, the validation data, used for determining when to stop the ANN training, and the test data will not be completely independent since it originates from the same subjects as data used for training. The test performance in Fig. 3(a) also indicates that there is a dependency between training data and test data as all ANNs display a very similar behavior. However, with the low complexity of our ANNs and the large amount of training data, there is no apparent risk for a substantial overtraining. This is also supported by Fig. 3(b) where the final evaluation, using completely untouched and independent data, display an average performance over all left-out subjects that is only marginally larger than the test performance [Fig. 3(a)].

Optical properties vary for different skin types and probably at different skin locations. In this study, we included only subjects with Fitzpatrick skin types I to III which exclude strongly pigmented skin types with larger fractions of melanin. The reference EPOS system can measure darker skin up to at least Fitzpatrick skin type V with good signal-to-noise ratio and model these spectra with good fidelity.^20^ However, the need for training ANNs to the full range of Fitzpatrick skin types I to VI is also indicated by recent observations in pulse oximetry, where darker skin results in a small but larger number of undetected hypoxemia events.^37^ Furthermore, all measurements were performed on the volar forearm during occlusion provocations. Our results suggest that a training data set of 19 subjects is enough for training an $S_{O_{2}}$ algorithm that can estimate $S_{O_{2}}$ in low-pigmented forearm skin. The system can probably be adapted to a variety of situations if an ANN, possibly using more than three hidden nodes, is trained on additional data covering also darker skin tones and other skin locations.

To evaluate how an ANN should be structured for our MSI system, we trained ANNs with different number of nodes in the hidden layer and executed the training 10 times for each size. Using the leave-one-subject-out procedure, ANNs were trained on 20 training data sets and performance of our method was estimated for 20 sets of evaluation data not included in the respective training data set. Using leave-one-subject-out results in a reliable and unbiased estimate of the model performance.^35^ The $E_{\mathrm{rms},W}$ in Fig. 3(b) varies only slightly for the 20 evaluation sets, and the minimum mean value indicates that a node size of 3 is sufficient to estimate $S_{O_{2}}$. Repeated trainings showed marginal differences, which implies a stable algorithm. Based on this, we concluded that repeated trainings were not necessary for our purposes. If the training is extended to other tissue types, sites, and/or provocation protocols, this may change. A larger number of hidden nodes could possibly distinguish more multifaceted and complex spectra but may also be overtrained which could cause overfitting to certain properties or parts of the spectrum. Furthermore, the algorithm’s computational time increases with an increased size of the hidden layer.

Bland–Altman analysis of $S_{O_{2},\mathrm{ANN}}$ compared to $S_{O_{2,\mathrm{EPOS}}}$ shows agreement at baseline and reperfusion, but slightly higher values of $S_{O_{2},\mathrm{ANN}}$ at end of occlusion. The result at reperfusion shows that relatively few training spectra with high target $S_{O_{2}}$ values does not affect performance when evaluating data with target values in that range.

As discussed in Ref. 2, the measurement depth is slightly different between MSI system and EPOS. Training ANNs with data from our MSI system to target EPOS $S_{O_{2}}$ values diminish those systematic differences. Different measurement sites probably explore and compare tissues with different physiology, including vascularization. Despite site differences this study shows that data from the probe-based reference system can be used as target and enable spatially resolved $S_{O_{2}}$ maps.

When trained ANNs are applied to MSI data used by Ewerlöf et al., results show agreement between $S_{O_{2},\mathrm{ANN}}$ and $S_{O_{2},\mathrm{invMC}}$ in the reperfusion phase but less conformance in baseline and at end of occlusion. ANNs were trained to fit EPOS measurements while the inverse MC algorithm was unconnected to data from the reference system. Therefore, a better agreement between EPOS and ANN is expected. Comparing images analyzed with ANN and inverse MC, respectively, show similar values for the three time points. The ANN algorithm results in a more homogeneous distribution of $S_{O_{2}}$ values. The computational time for the two methods is essentially different in favor of ANNs.

## 5 Conclusions

Our proposed ANN can estimate $S_{O_{2}}$ from data acquired by an MSI camera system with unknown spectral characteristics when it is trained to target $S_{O_{2}}$ values concurrently measured by a validated probe-based reference system. The high computational speed enables real-time imaging. Training data need to be chosen carefully to cover expected differences in $S_{O_{2}}$ and possibly also variation in skin type and skin location.
